# “The Wheel of My Work”: Community Health Worker Perspectives and Experiences with Facilitating Refugee Access to Primary Care Services

**DOI:** 10.1089/heq.2020.0150

**Published:** 2021-04-28

**Authors:** Nneze N. Eluka, Sharon D. Morrison, Holly S. Sienkiewicz

**Affiliations:** ^1^ Department of Public Health Education, University of North Carolina Greensboro, Greensboro, North Carolina, USA.; ^2^ Common Ground Health, Rochester, New York, USA.

**Keywords:** community health worker, qualitative study, refugee, primary care access, public health

## Abstract

**Purpose:** Community health workers (CHWs) are trusted community leaders and public health workers dedicated to promoting the health and well-being of community members. CHWs, who share similar language and culture, work with refugee communities that are often missed in traditional U.S. health systems. CHWs help refugees gain access to health care through culturally appropriate strategies. However, the scope of their study as cultural brokers with regard to refugee health access is largely unknown in the peer-reviewed literature. This qualitative research study used a constructivist grounded approach to examine the extent to which CHWs helped refugee clients gain access to the health care system.

**Methods:** Data were collected through interviews with a purposeful sample of 10 CHW participants affiliated with a primary care access program in Greensboro, North Carolina.

**Results:** The diagram derived from this study provided a schema that allowed for an improved understanding of CHW perspectives and experiences when connecting refugee clients to the health care system.

**Conclusions:** Further research incorporating CHW voices is recommended because CHWs are instrumental in improving the health and well-being of refugees.

## Introduction

Access to affordable and culturally responsive health care is the most frequently expressed need among recently resettled and longer term refugees.^1–6^ Access to care in the United States is defined in three stages: (1) gaining entry into the health system typically through health insurance coverage, (2) accessing a place for health services provision or geographic availability, and (3) accessing a health care provider with whom the patient is comfortable communicating or establishing a personal relationship.^7,8^ Unfortunately, refugees who attempt to navigate the complex U.S. health care system often encounter cultural, linguistic, socioeconomic, and structural barriers that limit their health care access.^2–4^ Even when low cost and culturally appropriate local health services are available, newcomers are often reluctant to utilize services due to anti-immigrant sentiments and shifting immigration policies that invoke fear of persecution and being barred from gaining citizenship.^9^ Yet, researchers collectively agree that increased primary care access contributes to improved health outcomes among this group, that is, a lower prevalence of chronic conditions such as diabetes, hypertension, cardiovascular diseases, some cancers, emotional distress, and other mental health conditions.^2–6^

Community health workers (CHWs) are a commonly used strategy for primary care access with vulnerable populations. In 1978, the World Health Organization (WHO) pushed for an increase in the utilization of CHWs for community level provision of essential health education and clinical services.^10,11^ CHWs are ideally members of the communities in which they work, knowledgeable about their communities' needs, and endorsed by the communities for their activities.^11^ They are often supported by the general health system, but not necessarily as a part of its organization, because they typically have less training compared with skilled health care professionals.^12^ This definition presents CHWs as unskilled workers with short and focused training that allows them to enter the workforce quickly.^13^ “Community health worker” is now used as a catch-all phrase for the ∼100 job titles that encompass CHW roles, for example, health promoter and lay health advisor.^14^

CHWs will increasingly be in demand to guide refugees new arrivals through navigating its complex health care system. Therefore, those refugee-serving organizations tasked with coordinating or administering CHW programming in resettlement contexts must be able to implement a seamless efficient CHW deployment process for positive health outcomes.^15,16^ Several studies have documented the refugee health access issues from the perspective of service providers,^17–19^ as well as refugee clients or service recipients themselves.^20–23^ However, fewer studies have examined the perspectives of CHWs working specifically with refugees.

Lipson et al. examined actual primary care access facilitation experiences of CHWs who were former refugees from Bosnia.^24^ These CHWs were interpreters who provided social support for recent refugee arrivals being deployed to cultural communities in the United States. They emphasized the importance of conducting thorough orientations within communities to (1) implement a more feasible “how to” process for navigating the U.S. primary care system and (2) present eligible households with accessible health care options. Finally, they leveraged their facilitation role to address a lack of language and culturally appropriate outreach programs specific to disease prevention, an important public health measure.

The mentioned study is illustrative of the importance of centering CHW perspectives and experiences within refugee health care access programming. This is critical because (1) we lack extensive documentation of how CHWs distinctively and differentially operationalize the job of connecting refugees to health services and (2) their shared perspectives is a gateway to better systematic allocation and deployment of resources for improved refugee health outcomes. Overall, insights from CHWs could inform efforts by program administrators and personnel responsible for postresettlement health promotion needs.^25,26^ Therefore, the purpose of this exploratory pilot study was to gather CHW perspectives and document distinct experiences with facilitating refugee adults' access to primary care, that is, local free clinics, health centers, private and hospital-affiliated general, internal, pediatric, and family medicine practices within a regional health system.

## Methods

Data were collected from 10 CHWs from April 2019 to October 2019 to examine their experiences of connecting with refugee community members and facilitating access to the primary care services in a metropolitan region in the U.S. south system. A purposive sample of CHWs was recruited through the Immigrant Health Access Project (IHAP), a locally funded initiative administered through the Center for New North Carolinians (CNNC). The CNNC is a service-focused center affiliated with the University of North Carolina at Greensboro. IHAP aims to eliminate language and cultural barriers in connecting eligible immigrant and refugee adults with primary care services in the Greater Greensboro area of North Carolina, a new settlement region for refugees and secondary migrants. Participants were 18 years and older, and were former and current CHWs with IHAP, or student interns at CNNC. All CHWs possessed language skills and refugee background and/or experiences. All CHWs received interpreter and client confidentiality training through a 2-day workshop at the CNNC. After completion, each CHW shadowed a seasoned CHW to observe the client interaction process. CHW participants were recruited by email, telephone, and in person. Each participant chose a pseudonym to protect their confidentiality. All participants provided verbal consent and were compensated $10 for participation. All study procedures were approved by the UNCG's Institutional Review Board.

A semistructured interview guide (Appendix A1) and a demographic questionnaire (Appendix A2) were developed from existing literature and with input from existing IHAP staff. The guide was used for key informant interviews with CHWs. Interviews were audio recorded, transcribed verbatim, and analyzed using constructivist grounded theory (CGT) methods. CGT takes into account a researcher's subjectivity and social location, past and present interaction with other people.^27^ CGT data analysis techniques include memo writing, coding, theoretical saturation, and constant comparison. Categories and analytic codes are developed such that pre-existing conceptualizations are not used.^28^

Each transcript was coded shortly after completing the interview. Insightful quotes were delineated and gerunds were used to describe each of these quotes. Memoing was also used to capture the thoughts and observations. After the lead author completed this initial coding process, they consulted with a qualitative methodologist. Together they reviewed this coding process and identified areas that needed recoding and would make the analyses stronger. Transcripts were recoded to compare differences and similarities across all 10 participants. Participant experiences were grouped and consolidated and themes assessed for similarities and uniqueness. Final memos were written to help explain the context of the emerging themes with the eventual grouping of recurrent themes.

## Results

The 10 CHW participants were representative of continental Africa and Asian regions. Their ages ranged from 19 to 45 years (average age being 30.5 years), with a majority (8) being female. Participant pseudonyms and profiles are presented in Table 1.

**Table 1.** Profile of Community Health Workers

Name (sex) age range	Type of CHW	Country of origin/region	Languages spoken	Experiences
Baba (M) 35–44	Full time	Central African Republic/Africa	Fula, Sango, French, Arabic	He has 9 years of experience working with the refugee community and 1 year as a CHW connecting them with the health care system. The primary populations he serves are Congolese and Sudanese. He has a college degree and has lived in the United States for ∼1 year. He is employed full time. He is a former refugee and worked in a refugee camp before being resettled in North Carolina.
Emma (F) 25–35	Full time	Vietnam/Asia	Jarai, Rhade, Vietnamese	She has 10 years of experience working with the refugee community and 1 to 2 years as a CHW connecting them with the health care system. The primary population she serves is Montagnards. She has a college degree and has lived in the United States for >10 years.
Savannah (F) 35–44	Full time	Vietnam/Asia	Jarai, Rhade, Vietnamese	She has >10 years of experience working with the refugee community and connecting them with the health care system. The primary population she serves is Montagnards. She has a college degree and has lived in the United States for ∼20 years.
Joan (F) 25–35	Contractor	Burundi/Africa	Swahili, Kinyarwanda, French, Kirundi	She has ∼2 years of experience working with the refugee community ∼1 year as a CHW connecting them with the health care system. The primary population she serves is Congolese. She has a high school diploma has lived in the United States for >5 years. She is a former refugee and worked in a refugee camp before being resettled in North Carolina.
Burmerican (M) 25–35	Contractor	Burma/Asia	Burmese, Nepali, Hindi	He has >2 years of experience connecting refugee community members with the health care system. The primary population he serves is Bhutanese. He has completed high school and has lived in the United States for >2 years.
Sue (F) 44+	Contractor	Vietnam/Asia	Vietnamese, Montagnard	She has 7 years of experience working with the refugee community and 1 to 2 years connecting them with the health care system. The primary population she serves is Montagnards. She has a medical degree from Vietnam and has lived in the United States for >10 years.
Heaven (F) 18–25	Contractor	Eritrea/Africa	Arabic, Tigrigna, Tigre	She has 3 years of experience as a CHW connecting refugees with the health care system. The primary population she serves is Arabic-speaking refugees. She is a student working on her college degree and has lived in the United States for >10 years. She is a former refugee.
Rachel (F) 25–35	Student intern	Rwanda/Africa	Swahili, Kinyarwanda, French	Rachel has 5 years of experience working with the refugee community and 1 to 2 years as an intern and CHW connecting them with the health care system. The primary population he serves is Congolese. She has a college degree and has lived in the United States for 10 to 20 years. She is a former refugee.
Prakriti (F) 18–25	Student intern	Nepal/Asia	Nepali, Hindi and Urdu	Prakriti has 7 years of experience working with the refugee community and 1 to 2 years connecting them with the health care system. The primary population she serves is Bhutanese. She has a college degree and she is a current student. She has lived in the United States for 10 to 20 years.
Barbara (F) 18–25	Student/AC member	Eritrea/Africa	Arabic, Tigrinya	She has 4 years of experience working with the refugee community and 1 to 2 years as an intern and CHW connecting them with the health care system. The primary population she serves is Arabic-speaking refugees. She is a student working on her college degree and has lived in the United States for 5 to 10 years. She is a former refugee.

CHW, community health worker; F, female; M, male.

**FIG. 1.**
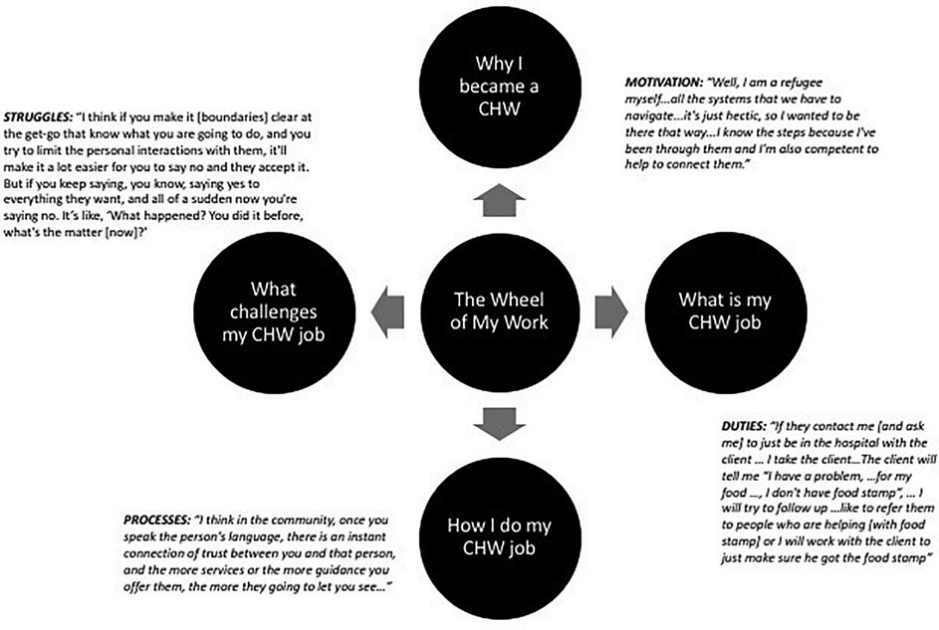
The wheel of my work.

Participants provided details on their motivations, roles, and duties as CHWs, with special focus on duties associated with refugee clients. These are reported under the following broad categories: why I became a CHW, what is my job, how I do my job, and what are the struggles associated with my job? They are organized within a “wheel of work,” See Figure 1.

### Why I became a CHW? (motivations)

Participants cited personal experience as a refugee as the main reason for becoming CHWs. For example, Barbara, an Arabic-speaking refugee from Eritrea, recalled arriving in the United States with no English proficiency. She struggled but eventually learned English and about U.S. social welfare and health care during AmeriCorps service. AmeriCorps is a voluntary service program administered by the U.S. government to engage adults in work that meets the needs of underserved populations and communities. She established a social support group for Arabic-speaking refugees:

Well, I am a refugee myself…all the systems that we have to navigate…it's just hectic, so I wanted to be there [for them] that way…I know the steps because I've been through them and I'm also competent to help to connect them.

Heaven was another Eritrean refugee whose decision to be a CHW was influenced by the cherished assistance her family received from refugee resettlement agencies and their supporting partners. She wanted to extend this same kindness and helpfulness to other refugees. Rachel, another refugee, had unrealistic expectations about life in the United States perceiving it as a “place where there is milk and honey.” She came to terms with a harsh reality that despite legal status, refugees were often prevented from accessing eligible resources (e.g., refugee Medicaid, food stamps, housing). Rachel became a personal guide for refugee clients to bridge this gap.

### What is my job? (duties)

There was variation in how participants interpreted their duties. Full-time IHAP CHWs and intern CHWs focused on resource connection for more distal health needs. Contract and student CHWs described their duties as helping clients with time-sensitive health issues. Emma and Baba are full-time CHWs. Emma categorized her main duty as *resource connection* to address community health needs. Baba's duties on the other hand centered on client support, problem solving, and case management. He explained:

…The client will tell me I have a problem, for example [with]my food stamp, “I don't have food stamp!.”… I will try to follow up with them… to refer them to people who are helping or I will work with the client to just make sure he got the food stamp because …it [food] has a lot of things related to health. So…I see it as case management… I will work to help him in every aspect of his life.

Emma and Baba interpreted their roles more holistically to include social support services.

Heaven and Burmerican represented contract CHWs who typically spend a few hours a week at community centers located within refugee-dense apartment housing complexes. They described their CHW duties as *client assistance* (e.g., medical billing, connecting them to a primary health care home), resource connections, and interpretation. Burmerican typically gave an informal introduction to community members:

There is not a term for CHW in Burmese and Nepali [langauges]. I say that I help to…navigate the healthcare system. I always assume [role] that I am an interpreter. The terms give people an idea of what I am.

The student CHWs (typically interns with IHAP) completed service hours for their bachelor's degree in a health and/or human service program. Prakriti, Rachel, and Barbara, as student CHWs, collectively categorized duties as being “fluid” with focus on advocacy. All participants saw themselves primarily as a resource person for refugee clients assisting with health insurance access, service provider appointments, and health education needs. CHWs explained how to take their medication correctly or how to refill their prescriptions. CHWs also taught clients how to independently negotiate care visits and converse about personal health with service providers.

### How I do my job? (processes)

Participants identified two key processes—communication and trust building—as critical to their CHW duties. The communication process necessitated partnerships longer standing clients. Some CHWs were physically located at the community centers at apartment complexes for face-to-face interaction with clients. This involved group and door-to-door visits for informal introductory conversations with new clients about the services CHWs could provide. CHWs also had follow-up discussions with established clients through planned home visits and gained new clients through word-of-mouth referrals. With time, CHWs were perceived as approachable, with communication loops that built rapport and allyship among refugee households.

CHWs leveraged their cultural broker role to build trust with clients. For example, Barbara indicated that sharing a common language opened the door for a trusting working relationship with clients.

I think in the community once you speak the person's language, there is an instant connection of trust between you and that person, and the more services or the more guidance you offer them, the more they going to let you see, and sometimes they're just so…they want, need that assistance, so anybody who is willing to help, they will give them the time.

### What are the challenges to my job? (struggles)

Although CHWs felt equipped to handle their CHW duties, they encountered unique challenges. They were challenged with setting boundaries to maintain professionalism with clients with whom they had strong relationships. The flexibility of their duties often led to clients burdening and overwhelming them with needs. Barbara explained that clients sometimes wanted her to become “their personal caseworker” and essentially assigned her duties beyond her CHW role:

I think if you make it clear at the get-go what you are going to do, and you try to limit the personal interactions with them. It'll make it a lot easier for you to say no and they accept it. But if you keep saying, you know, saying yes to everything they want, and all of a sudden now you're saying no. It's like, “What happened? You did it before, what's the matter?”

CHW also struggled with frustration when best efforts to assist clients did not always work out as planned. CHWs viewed health insurance as a tool to connect clients along a cascade to help improve their health and well-being. However, they also felt frustrated while connecting clients with health insurance at a systemic level. Owing to the variability in health insurance coverage, some refugees did not have full coverage and sometimes needed to pay for health services out of pocket after the 8 months refugee Medicaid ended. For example, Sue explained that she felt bad because her attempts to connect her clients with a primary care doctor sometimes resulted in them getting stuck with large bills due to limited Medicaid coverage.

The thing is that when we talk about health, we encourage them [to] establish [a] family care doctor and then we interpret [for them], and then after that, the bill come[s] back and that frustrate[s] me… When the bill comes back… I feel bad that… I'm the reason [they] have to owe something…

## Discussion

This exploratory pilot study examined perspectives and experiences of CHWs in connecting refugee clients to local primary care services within a larger regional health care system. Results revealed factors that dictated the scope of CHW role and duties and are represented within a CHW “wheel of work.” See Figure 1. Central to this wheel were motivations, duties, processes, and struggles, four themes that emerged from CHW perspectives and experiences when facilitating refugee access to primary care. Collectively, CHWs in this study were highly motivated to perform their roles. These CHWs gravitated toward helping their community out of a sense of duty, despite the many struggles and challenges they knew awaited them in the CHW role.

Swartz and Colvin suggest that the work of CHWs is complex and intertwines with an overlapping narrative of motivations that must be considered within the context where and how they work.^29^ CHWs in this study worked in a mixed market environment with a combination of paid and unpaid CHWs. CHWs in both groups were motivated by the perceived benefits from being dedicated and committed to providing service to their communities. However, these same CHWs can easily become demotivated in the absence of adequate support, preparation and training from outreach supervisors or program managers.^30^ Although this study did not focus on demotivation, this is an important challenge to the CHW role and duties associated with refugee health access that warrants further attention.

The CHW processes uncovered as part of the “wheel of work” in this study were instrumental in facilitating refugee access to health information and primary care services. The CHWs employed processes that relied on clear and culturally congruent communication strategies, for example, face-to-face or door-to-door conversations using common cultural languages and invoked patience and honest empathetic insider dialog as part of the trust-building process. These “soft skill” processes provide a gateway for refugees to engage and utilize CHW services in times of need. Previous studies have confirmed CHWs' ability to leverage these processes engage with minority groups in a manner that outsider institutions and persons are not able to.^24,31^ Furthermore, CHWs navigate between using prescribed procedures and applying unique personal skills that are characterized by a high degree of discretion and informality.^32^

In this study, we did not find any evidence of Lehmann and Sanders' differentiation between generalists and specialists.^33^ In fact, all CHWs involved could be seen as generalists because they actively helped refugee clients get connected to a range of primary care services using similar mechanisms. However, for some, their duties extended beyond this because they provided resources to meet distal health needs. Finally, all CHWs identified struggles. These included boundaries to maintain professionalism with clients, and handling frustration and disappointment in the wake of negative outcomes for their clients. These issues may be absent from CHW training, yet represent important challenges that CHWs encounter and must tackle while working with clients.

Although this study yielded important information about the nuanced nature of the CHW experience and work related to refugee access to primary care, it is not without its limitations. First, the lead author interviewed the CHWs from the most populous language groups served by the CNNC. There are, however, smaller refugee populations that have received CHW services. CHW perspectives and experiences from working with these groups were not represented in this study. Yet, these may provide important insights into how CHW operationalize their duties to meet refugee client needs. Second, English was not a first language for any CHW. All acquired English through informal or formal means. Thus, their comfort level with expressing nuanced cultural concepts or issues may have limited the comprehensiveness of their experiential descriptions. Third, the follow-up questions may have been more intensive with individuals whom the first author had interacted with for a longer duration due to privileged knowledge of their background and experience compared with others whom the first author had interacted with the least. However, the interview guide helped to frame the questions asked and helped to ensure a level of uniformity across interviews. Fourth, CHWs may have participated because the first author had established trust with them through her work as a graduate research assistant working closely with the director of the CNNC. This may have influenced participants' willingness to speak candidly about negative experiences during current or immediate past affiliation with the CNNC. This information was necessary in providing a balanced picture of their work. Finally, the majority of CHW participants were females. To gather more in-depth insights into the gendered nature of the CHW work with refugees, more males should be included in future studies.

## Conclusion

The findings from this study are particularly important because CHWs continue to be instrumental paraprofessionals who play a key role in improved health of refugee communities. These can be used to tailor future interventions for training and supporting CHWs working with refugee populations in the United States. They can assist in better defining promising practices to reduce existing health burdens and promote positive outcomes in refugee communities.

## Abbreviations Used

CHWs: community health workers
CGT: constructivist grounded theory
CNNC: Center for New North Carolinians
IHAP: Immigrant Health Access Program
WHO: World Health Organization

## Appendix

### Appendix A1. Demographic Profile Questionnaire

What is your age group? □18–24 □25–35 □35–44 □44+

What is your sex/gender? □Male □Female □Other/prefer not to answer

Are you current or former IHAP CHW? □current □former

How long have you worked with refugees? ________less than 1 year _________years

How long have you worked as an IHAP CHW? □1–2 years □3–5 years □6–10 years □10+ years

Which languages do you speak?

What is your country of origin?

What is your highest level of education? □Less than high school □High school diploma □College degree □Higher than college degree

How many years have you lived in the United States? _____years

Outside IHAP, do you work: □Part-time □Full-time □Student □Not employed/Other

### Appendix A2. Individual Interview Guide

(1) From my experience studying community health workers (CHWs), I have found that there is a lot of confusion about what CHWs actually do! How do you introduce yourself as a CHW to people in the refugee communities that you serve?
(2) How do you connect with refugees who are new in the community?
(3) Starting from the beginning, can you describe the steps you would take when working with a new client?
(4) How about during a second or third contact with a client, what type of services would you provide after the first contact?
(5) Can you tell me more about how you would follow up with clients after you have helped them to connect with the health care system?
(6) There are a lot of success stories that have been recorded with CHWs working with refugee clients. Can you reflect a bit on your own experience as a CHW and please share with me one success story from your experience?
(7) Thank you for sharing that. How did you feel about your role in creating that success story?
(8) Let us now turn to barriers, because there are success stories alongside barriers in the CHW field. Can you reflect on your experiences and please share one barrier that you have experienced?
(9) How did you handle or navigate that barrier?
(10) You mentioned earlier that you have worked with refugees for (length of time) and been a CHW for (length of time). What events led to you becoming a CHW?
(11) Tell me about the strengths or skills that you have developed or discovered through serving as a CHW?
(12) Given your CHW experience, what advice would you give a new CHW? And why?
(13) Is there something else you think I should understand about being a CHW?
(14) Is there anything you would like to ask me?
